# Platelet-Rich Plasma in Treatment of Zoledronic Acid-Induced Bisphosphonate-related Osteonecrosis of the Jaws

**DOI:** 10.5812/traumamon.17196

**Published:** 2014-03-18

**Authors:** Farzin Sarkarat, Mohammad Hosein Kalantar Motamedi, Jahanfar Jahanbani, Dena Sepehri, Roozbeh Kahali, Zahra Nematollahi

**Affiliations:** 1 Department of Oral and Maxillofacial Surgery, Buali Hospital, Azad University of Medical Sciences, Tehran, IR Iran; 2 Trauma Research Center, Baqiyatallah University of Medical Sciences, Tehran, IR Iran; 3 Department of Maxillofacial Surgery, Azad University of Medical Sciences, Tehran, IR Iran; 4 Department of Oral and Maxillofacial Pathology, Dental Branch, Azad University of Medical Sciences, Tehran, IR Iran; 5 Private Practice, Tehran, IR Iran; 6 School of Dentistry, Shahid Beheshti University of Medical Sciences, Tehran, IR Iran

**Keywords:** Zoledronic Acid, Bisphosphonate, Osteonecrosis, Bisphosphonate-Related Osteonecrosis of the Jaw, Osteoporosis, Platelet-Rich Plasma

## Abstract

**Background:**

Bisphosphonate-related osteonecrosis of the jaws (BRONJ) is a well-known challenging entity warranting management. Platelet-Rich Plasma (PRP) plays an important role in bone biology by enhancing bone repair and regeneration.

**Objectives:**

The aim of this animal study was to evaluate the effects of PRP on zoledronic acid-induced BRONJ.

**Materials and Methods:**

Seven rats were given 0.04 mg Zoledronic acid intravenously once a week for five weeks. Two weeks later, the animals underwent extraction of their first lower molars, bilaterally. After clinical confirmation of the osteonecrosis, PRP was injected randomly into one of the extraction sockets of each rat. Three weeks later, all rats were sacrificed in order to obtain histological sections. The analysis of epithelialization was performed by McNamar’s test, and the analysis of osteogenesis and angiogenesis was performed by the Wilcoxon Sign Rank test. P value was set at 0.05.

**Results:**

We found no significant differences between the two groups regarding the amount of epithelialization, angiogenesis or sequestrum formation (P > 0.05), but a significant difference was seen between the two groups regarding the amount of existing vital bone (P < 0.05).

**Conclusions:**

Our study demonstrates positive results (preservation or regeneration of bone) using PRP in treatment of BRONJ. Although PRP may enhance osseous regeneration, long-term follow-ups are required to confirm its benefits.

## 1. Background

Bisphosphonates (BPs) are stable analogs of inorganic pyrophosphate, which are well-established anti-bone-resorption drugs used for over 30 years (1); however, their specific mechanism of action is still unclear (2). BPs are classified into two groups: non-nitrogen-containing (Etidronate, Clodronate) and nitrogen-containing BPs (Pamidronate, Residronate, Alendronate, Zoledronate or Zoledronic acid) used in treatment of some pathologic conditions such as hypercalcemia, Paget's disease, postmenopausal osteoporosis, bone metastasis and multiple myeloma (1, 3-6). BPs induce bone turnover suppression, inhibit the ability to repair bone microdamages, increase bone mineral density, induce osteoclast apoptosis, stimulate osteoclast inhibitory factors, and inhibit osteoblastic function and osteoclast differentiation from monocytes. They are also anti-angiogenic, and in theory, their ability to inhibit angiogenesis and vasculogenesis may be accentuated in bones with high vascularity and bone turnover, such as the jaw bones (2, 7, 8).

Bisphosphonate-related osteonecrosis of the jaws ( BRONJ) is a well-known adverse effect of long-term bisphosphonate therapy, not only representing a challenge for the dentist and the maxillofacial surgeon but also for the oncologist and the physician (8). BRONJ is defined as an avascular area of necrotic bone with or without exposure in the maxillofacial region that does not heal within 6-8 weeks in a patient who received Bisphosphonate therapy with no history of radiation therapy to the craniofacial region (3, 8-10). The incidence of BRONJ is two-folds greater in the mandible (77%) compared to the maxilla and more in women (72%) compared to men (11). Studies have identified various risk factors such as type of BPs and duration of exposure to them, type of malignancy, metastasis, chemotherapy, obesity, etc., which are associated with the development of BRONJ (7-9).

Currently, BRONJ management remains controversial and there is no definite standard care. Based on clinical staging, treatment of BRONJ has varied from clinical approaches such as 0.12% chlorhexidine gluconate mouthwash and oral systemic antibiotics to major local surgical debridement. Surgery is recommended in patients who are symptomatic, such as those with pathologic mandible fractures or have necrotic bone as a source of infection or patients who do not respond to conservative treatments (2, 12-19). There are many studies implicating the role of different cellular mediators bone morphogenic protein and angiogenic growth factors in the healing process of bone defects (2, 20-32). Platelet-Rich Plasma (PRP) is a concentration of growth factors such as platelet-derived growth factors, transforming growth factor-β, vascular endothelial growth factor, epidermal growth factor, insulin-like growth factor (2, 31-33) and also osteoconductive proteins which can play a major role in bone biology by accelerating and enhancing bone repair or regeneration (34).

## 2. Objectives

This paper describes the results of using PRP in the management of BRONJ induced by zoledronic acid in rats.

## 3. Materials and Methods

This study was conducted in the oral and maxillofacial surgery department of our university.

### 3.1. Experimental Design

At the beginning of this interventional animal study, we selected seven female rats, which were free of infection or pathologic conditions interfering with the experiment. We kept the rats in large cages at a temperature of 20 ± 0.5˚C, 55 ± 10% humidity with food and water ad libitum.

For this split mouth study, rats’ teeth were divided equally into control and experimental groups. All rats received intravenous injection of 0.2 mL diluted Zoledronic acid (0.04 mg Zoledronic acid in 0.2 mg/mL normal saline) once a week for a total of five weeks.

### 3.2. Protection of BRONJ

After two weeks, the mandibular first molars of all rats were extracted bilaterally under general anesthesia by the intramuscular administration of 50 mg/kg Ketamine (Ketaminol Vet, Veterinaria AG, Zurich, Switzerland) and 8 mg/kg Xylazin (Rompun Bayer AG, Leverkusen, Germany). After clinical confirmation of osteonecrosis, 0.15 mL PRP was injected randomly into one of the two extraction sockets of each rat.

### 3.3. PRP Preparation

The PRP (Rooya Gen PRP kit, Arya Mabna Tashkhis Co., Tehran, Iran) used in this study was obtained as follows: 

First, two milliliters of blood were taken from each sample using PRP kit tubes containing 0.3 mL of anticoagulant and separator in order to prevent blood coagulation. After transferring the tube contents to another tube containing platelet activation preventing substances and centrifugation of the tube at 1700 rpm for 12 minutes, its upper component, which contained platelets and plasma was transferred to another tube containing preservative substances. Then, the tube was centrifuged at 3500 rpm for seven minutes. Finally, based on the manufacturer’s instructions, the upper portion of the tube contents was discarded and after 30 minutes delay, the remaining portion, which was rich in growth factors, was injected into the extraction sockets.

Three weeks later, after clinical confirmation of epithelialization of the sockets, the animals underwent another general anesthesia as described above, and then sacrificed. Their jawbones were removed, and kept in 10% formalin solution.

After 10 weeks, the specimens were demineralized by using EDTA 5%/ formalin 10% solution for 10 weeks. Several longitudinal frontal sections (5 µm width) were obtained from the samples buried into paraffin, and then they were prepared for histopathological assessment after staining with haematoxylin-eosin (H&E). Blood vessels, bone sequestrae and vital bone patterns were investigated using a light microscope (Euromex, Arnhem, Holland) at 100× and 400× magnifications with Image Focus software 2009 version 25.

### 3.4. Statistical Analysis

The evaluation of the epithelialization was performed by the McNemar’s test and evaluation of osteogenesis and angiogenesis was performed by the Wilcoxon Signed Rank test using SPSS software (version 18). P value was set at 0.05.

## 4. Results

At the onset, seven rats were included in this study. Unfortunately, one of them died during the study; thus, statistical analysis was performed on the data collected from the six remaining rats.

The rats were of the same age, sex, environmental and treatment conditions. Table 1 shows the main data corresponding to epithelialization, angiogenesis, sequestrum formation and existing vital bone.

**Table 1.** Comparison of Angiogenesis, Sequestrum Formation and Existing Vital Bone in Experimental and Control Groups ^a^

	Experimental	Control	P value
**Angiogenesis**	23.33 ± 5.16	25.00 ± 5.47	0.65
**Sequestrum formation**	3.33 ± 5.16	8.33 ± 4.08	0.18
**Existing vital bone**	75.83 ± 16.25	48.33 ± 2.58	0.03

^a^ Data are presented as Mean ± SD, %.

There were no significant statistical differences in epithelialization, angiogenesis and sequestrum formation between the experimental and control groups (P > 0.05). However, a significant difference was found relative to the amount of existing vital bone between the experimental and control groups (75.83 ± 16.25 % and 48.33 ± 2.58%, respectively, P < 0.05).

## 5. Discussion

The efficacy of Bisphosphonates in reducing the symptoms and complications of bone diseases has been extensively documented (35-41). Among Bisphosphonates, Zoledronic acid is a chemical compound frequently associated with BRONJ, due to its major pharmacological efficacy and its widespread use (1, 42). It acts on osteoblasts, stimulating the release of osteoclast recruitment inhibitors. Furthremore, Zoledronic acid demonstrates anti-angiogenesis effect through inhibition of endothelial cells, by reducing proliferation and inducing apoptosis (34).

BRONJ is an important complication in some patients receiving this class of drugs with an incidence of 0.8% to 12% per year (1, 17, 35, 43). In most cases, BRONJ follows an oral surgery procedure, usually dental extraction, which is the cause of 40-80% of BRONJ cases.

PRP was first introduced by Marx, in combination with autologous bone grafts for the reconstruction of mandibular defects (1, 21).

No universally accepted therapeutic protocol is known to eradicate BRONJ, as its etiopathogenic mechanism remains unclear; however, the treatment goal should be focused on eliminating pain and preventing progression of bone infection and necrosis (34).

In this study, we investigated the effect of PRP on treatment of Zoledronic acid-induced BRONJ's angiogenesis, epithelialization and sequestrum formation in rats. The obtained results showed no significant differences in angiogenesis and sequestrum formation between samples; although, the amount of vital bone was significantly greater in the group treated with PRP.

Bocanegra-Perez et al. and Scoletta et al. proposed using PRP in a surgical protocol to reduce occurrence of BRONJ in patients under IV Bisphosphonate treatment who required exodontia (34, 44).

Bocanegra-Perez et al. and Yokota et al. observed accelerated angiogenesis of necrotic bone in rabbits by combining vascular tissue and a single PRP injection (34, 45). In a study on rabbits, Aguirre et al. and Lopez-Jornet et al. stated that the use of Plasma Rich in Growth Factors (PRGF) accelerates epithelialization and reduces inflammation in wounds of the tongue (1, 46). Our results confirm this result. Curi et al. studied 25 patients with BRONJ with a history of intravenous Bisphosphonate therapy for metastatic bone diseases; they were treated with a combination of necrotic bone resection and PRP. They found satisfactory results of complete wound healing in most cases (80%) and a shorter BRONJ treatment period (2). In Mozzati et al. study on 32 BRONJ patients, treatment was performed by resection of the necrotic bone and PRGF use (3).

Contrary to Curi et al. results, Mozzati et al. and Coleman et al. stated vascularization and regeneration enhancement of both osseous and epithelial tissues; however, we did not obtain this result in our study. This difference may be due to non- existing necrotic bone because they resected the bone in their study (3, 4). We did however, note enhancement in the amount of vital bone in our study. Bocanegra-Perez et al. used PRP in the surgical treatment of the eight BRONJ patients. All patients improved within three weeks after treatment with fast mucosal healing, reduced need for analgesic and better resolution of mouth lesions (34).

Cetiner et al. described a case of Zoledronate-associated BRONJ after tooth exodontia in a 68-year-old man with multiple myeloma treated with surgical debridement plus PRP, showing a good outcome after six months follow-up (34, 47). It should be noted that PRP pH value ranges between 6.5 and 6.7, which is more acidic than blood (7.0 - 7.2), and therefore it is expected to be less favorable for bacterial growth (27, 34). PRP use should be avoided in patients with precancerous lesions or with a history of Squamous Cell Carcinoma.

Even though our analyzed sample size was small, the benefits described showed the use of PRP had favorable results in treatment of BRONJ, especially in cases that need to enhancement of the healing process. More studies with longer follow-up are recommended.
